# Around the Hospitals

**Published:** 1893-10-14

**Authors:** 


### Around the Hospitals.

Notice to the Managers of Institutions.—Special space is now reserved for the insertion of notices of hospital meetings and festivals. The Editor requests that all notices may reach The Hospital Office, 428, Strand, at least one week before the date of each meeting.

**The Victoria Hospital, Swindon.**—The committee of the Victoria Hospital, in presenting their fifth report, are able to show that the affairs of the hospital generally are in a prosperous and satisfactory condition, and that the work done during the past year has met with due appreciation amongst all classes of the community. It has been abundantly proved that by its establishment a want has been supplied in the district. The necessity for such a centre of medical aid is seen by a glance at the list of urgent cases, accidents, and otherwise which have been attended to during 1892. The fact that the Great Western Railway Company's engine works, with their many hundreds of employés, are situated at Swindon, is an additional reason for its existence. Some 66 patients were admitted and treated during the year. Various improvements have been effected, in the heating apparatus and in repairs, and repainting, &c., but we do not notice any mention of certain necessary alterations in the matter of drainage, which we hope have, however, been carried out. The building and furnishing account shows an increase, and has reached the sum of £435 5s. 7d., of this the committee specially acknowledge a donation of £21 from Mr. Edgar Hanbury, and £67 16s. 3d. from the Hospital Saturday Fund. The services of the medical staff and of the matron and nurse receive special mention in the yearly report, and it is pleasant to note that the many friends of the hospital have not been backward in gifts of game, fruit, and other useful contributions in kind. Old linen and calico are thankfully received and much needed. We notice that it is proposed to alter and amend certain of the rules in order to facilitate the admission of patients who live some distance from any member of the medical staff.

**Central London Throat and Ear Hospital.**—During the past year the committee of this hospital have taken advantage of an opportunity of securing some adjoining land for much-needed additional accommodation. This has been done with the aid of a loan of £5,000 from the bankers, and the committee are now doing their utmost to obtain a further £5,000 for the erection of the necessary buildings. This increase of space is sorely wanted, seeing that the present report shows that "the accommodation for the treatment of 173,821 out-patients in the last five years is precisely the same as that which was available in the first five years of the hospital's existence, when only 72,660 patients were treated. The same observation applies to the in-patients." It is proposed that the new buildings shall comprise out-patients' departments, isolation wards, a recreation ground, and day rooms for the in-patients, and improved and more suitable rooms for the nursing staff and the servants. The system of payment by patients according to means obtains at this hospital with satisfactory results, though destitute cases are admitted freely and without the necessity of seeking a letter of recommendation. We notice that the Duke of Connaught has consented to become a patron, and has contributed £20 in aid of the funds. A building fund is open for the objects to which we have alluded above, and donations towards the balance of £5,000 are urgently appealed for by the committee.

